# Transcriptomic and targeted metabolomic analysis identifies genes involved in differential anthocyanin accumulation in potato tubers

**DOI:** 10.3389/fpls.2025.1615972

**Published:** 2025-06-19

**Authors:** Rui Yang, Shuohan Huang, Dan Li, Yuan Sun, Guangdong Zhou, Duanrong Zhou, Binquan Huang

**Affiliations:** State Key Laboratory for Conservation and Utilization of Bio-Resources in Yunnan, School of Agriculture, Yunnan University, Kunming, China

**Keywords:** potato tuber, anthocyanin, transcriptome, metabolome, regulatory network, StWRKY44

## Abstract

Purple-fleshed potatoes accumulate high amounts of anthocyanins, which are beneficial to human health. Although the biosynthesis of these secondary metabolites has been well studied in plants, the mechanisms underlying anthocyanin accumulation in different tissue regions of potato tubers remain less understood. To identify genes and metabolites involved in anthocyanin accumulation, we performed comparative transcriptomic and metabolomic analyses of pith and vasculature tissues from the tubers of three different potato cultivars. Anthocyanin-targeted metabolome analysis revealed that 20 anthocyanins were key metabolites conferring purple pigmentation in the tuber. Integrated transcriptomic and metabolomic analysis identified 1,924 genes potentially involved in multiple pathways for the biosynthesis of these anthocyanins. In particular, we identified 47 genes that were specifically expressed in the tuber and highly correlated with different anthocyanins. These genes were associated with ATP-binding cassette transporters and phytohormone pathways. Additionally, a core transcription factor, StWRKY44, involved in anthocyanin accumulation in the tuber was identified; it was capable of binding to and activating the promoters of 7 anthocyanin structural genes. This study provides insights into the genes and metabolites underlying anthocyanin accumulation in potato tubers, which will be valuable for future functional studies and breeding efforts.

## 1 Introduction

Potato (*Solanum tuberosum* L.) is the most important non-grain food crop species in the world (http://www.fao.org). It has become an indispensable crop for meeting human dietary needs. While most potato cultivars exhibit white or yellow flesh, some varieties display purple flesh due to the accumulation of elevated levels of anthocyanins. Anthocyanins, a class of important flavonoid pigments in plants, possess strong antioxidant activities and other bioactive properties that are beneficial to human health (Pojer et al., 2013). Thus, understanding the molecular mechanisms of anthocyanin biosynthesis in potato tubers could contribute to the development of health-promoting products.

Anthocyanins are synthesized through the phenylpropanoid pathway, a conserved biochemical route that begins with the conversion of phenylalanine to cinnamic acid by phenylalanine ammonia-lyase (PAL). Subsequent enzymatic steps involve chalcone synthase (CHS), chalcone isomerase (CHI), flavanone 3-hydroxylase (F3H), and dihydroflavonol 4-reductase (DFR), which together lead to the formation of leucoanthocyanidins. Anthocyanidin synthase (ANS) catalyzes the oxidation of leucoanthocyanidins to anthocyanidins, which are further glycosylated by UDP-glucose anthocyanidin 3-O-glycosyltransferase (UFGT) to form stable anthocyanins. These pigments are transported to vacuoles via glutathione S-transferases (GSTs) or membrane-bound transporters, where they accumulate in subcellular compartments (Huang et al., 2024; Jaakola, 2013). Structural genes encoding these enzymes are spatially and temporally regulated, with tissue-specific expression patterns observed in flowers, fruits, and stress-responsive organs.

The spatiotemporal biosynthesis of anthocyanins is tightly controlled by transcription factors (TFs), predominantly the MYB-bHLH-WD40 (MBW) complex. R2R3-MYB TFs, such as *AtMYB75*/*PAP1* in *Arabidopsis* and *MdMYB10* in apples, directly bind to promoters of structural genes to activate their expression (Espley et al., 2007; Shin et al., 2013). Conversely, the anthocyanin biosynthesis pathway is also controlled by MYB repressors, which act upon MBW complexes or directly bind to the promoter of target genes to form a complex regulatory network of anthocyanin biosynthesis in plants (Chen et al., 2019). bHLH proteins can interact with MYB factors to enhance anthocyanin production (Li et al., 2022b; Quattrocchio et al., 2006), while WDR serves as a protein–protein interaction platform to facilitate the formation and stability of the MBW complex (Lloyd et al., 2017; Vander Kooi et al., 2010; Zhu et al., 2024). In addition to the MBW complex, TFs from other families play crucial roles in the biosynthesis of anthocyanins, such as *WRKY44* (Peng et al., 2020), *WRKY70* (Zhang et al., 2024), *WRKY75* (Lu et al., 2024), *ERF9* (Ni et al., 2023), *NAC002* (Zou et al., 2023), *bZIP9* (Chen et al., 2024). Such a multilayered regulatory network ensures precise control over anthocyanin levels in response to developmental and environmental cues.

Phytohormones play pivotal roles in regulating anthocyanin biosynthesis. Abscisic acid (ABA) can promote anthocyanin accumulation by activating the expression of structural genes and enhancing the function of the MBW complex, such as TFs *ABI5* (Song et al., 2024). Jasmonic acid (JA) induces JAZ5/10 degradation, thereby promoting the stability and transcriptional activity of the MYB5-TT8 complex and enhancing anthocyanin biosynthesis (Li et al., 2024). In the presence of gibberellins (GA), the activities of MBW complexes are inhibited by MYBL2 and/or JAZ repressors and suppress the anthocyanin biosynthetic pathway (Xie et al., 2016). The auxin indoleacetic acid (IAA) exhibits complex dose-dependent effects on anthocyanin synthesis, with high concentrations often inhibiting and low concentrations promoting anthocyanin production (Wang et al., 2018). Collectively, these plant hormones and TFs form a complex regulatory network that fine-tunes anthocyanin biosynthesis, enhancing plant adaptability to various stress conditions, such as temperature, light, salt stress, and drought (Li et al., 2022a, 2016; Liu et al., 2024; Wang et al., 2024).

Although anthocyanin metabolism has been well studied in aboveground plant organs (e.g., flowers, fruits, and leaves), the mechanisms underlying anthocyanin accumulation in the underground tuberous stems of potatoes are less understood (He et al., 2024). We conducted a comparative analysis of the transcriptome and anthocyanin-targeted metabolome data derived from the tubers of the Qicai, Heijingang, and Mila cultivars to gain a better understanding of the molecular and metabolic mechanisms of anthocyanin pigmentation in potato tubers. New candidate genes participating in different anthocyanin deposition and contributing to metabolite flux divergence in the tuber were identified. This analysis revealed genes and key metabolites that contribute to anthocyanin pigmentation in the tubers of potatoes, providing new insights for further elucidation of the underlying networks in this non-grain food crop species.

## 2 Materials and methods

### 2.1 Plant materials and sampling

**Figure 1:**
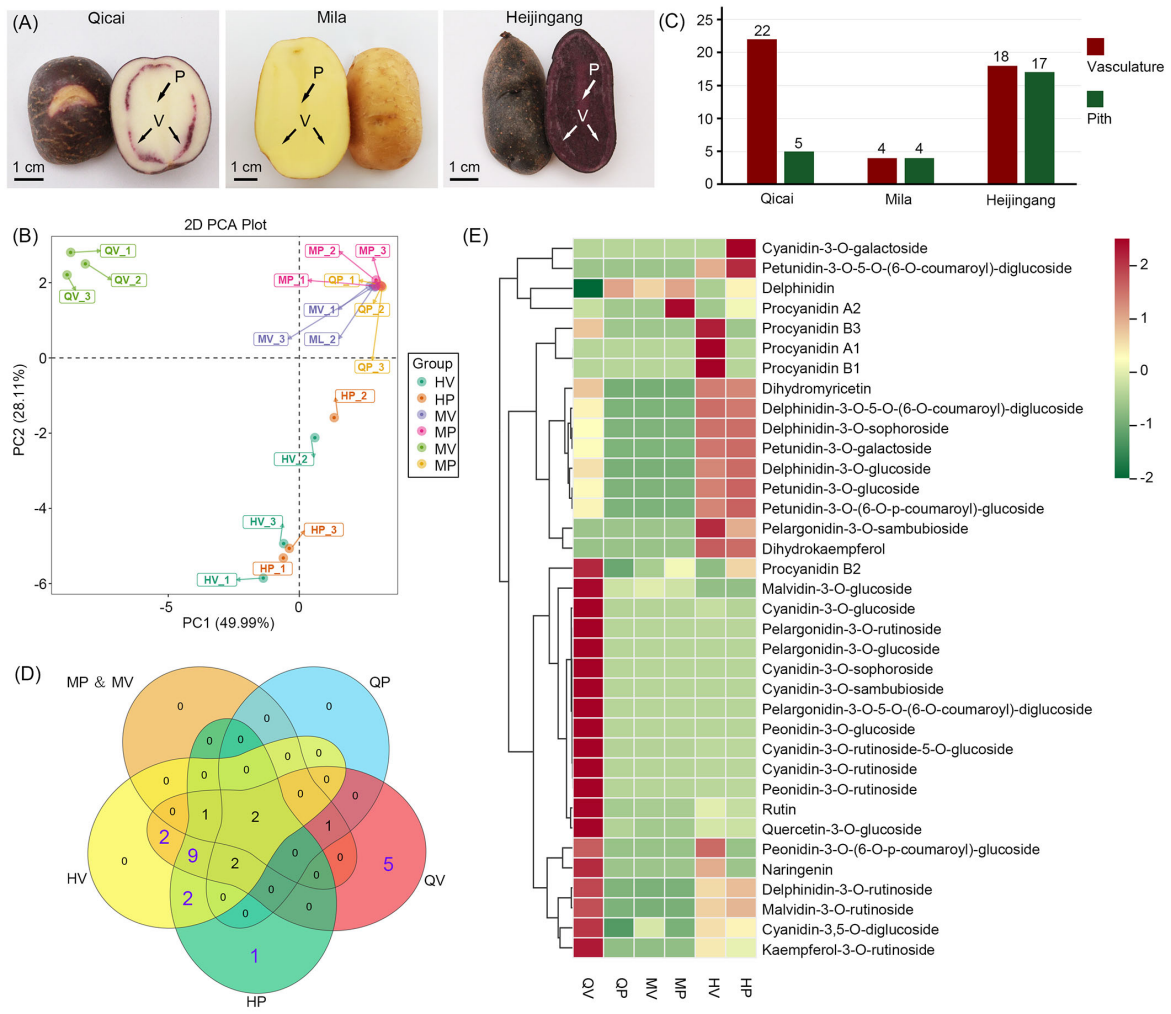
Morphology of tuber flesh with different purple tubers and an overview of the metabolome profile in purple tubers. **(A)** Tuber flesh colors of three different potato cultivars: Qicai, Mila, and Heijingang. P, pith; V, vasculature. **(B)** Principal component analysis (PCA) of anthocyanin contents identified in all samples. MP, Mila pith; MV, Mila vasculature; QP, Qicai pith; QV, Qicai vasculature; HP, Heijingang pith; HV, Heijingang vasculature. **(C)** The column diagram shows the numbers of anthocyanin monomers in Qicai, Mila, and Heijingang. **(D)** Venn diagram showing 25 anthocyanin monomers across different potato varieties and tissues. **(E)** Heat map showing the relative contents of anthocyanin metabolism-related compounds. For each compound, the normalized content values were calculated by the maximum value of all compound content levels for six different samples. The color scale was normalized to range from −2 (green) to +2 (red), which represented the high and low expression values, respectively.

Three tetraploid potato cultivars representing different tuber flesh colors (Qicai, Mila, and Heijingang; **Figure 1A**) were propagated *in vitro* using single-node stem on Murashige-Skoog medium supplemented with 3% (w/v) sucrose for 2 weeks under long-day (LD) (16 h of light and 8 h of darkness) conditions at 22°C. The plants were then transferred to the soil and maintained in a climate chamber for 3 weeks under LD conditions. After 3 weeks under LD, plants were transplanted into plastic pots with a diameter of 25 cm (one plant per pot) in a growth room with a short-day (SD) photoperiod (8 h of light and 16 h of darkness) at 20 ± 2°C and were managed to ensure normal growth. Tubers were harvested at a fully mature (two-month-old) stage following transcriptomic and metabolomic analyses. Flesh tissues were immediately cut into pieces after harvest, frozen in liquid nitrogen, and stored at −80°C until analyses. Three biological replicates were prepared for each sample.

### 2.2 Anthocyanin-targeted metabolome analysis

Anthocyanin contents were detected using MetWare (http://www.metware.cn/) based on the AB Sciex QTRAP 6500 LC-MS/MS platform. The hierarchical cluster analysis (HCA) results of the samples and metabolites were presented as heatmaps with dendrograms. HCA was conducted using OmicShare Analysis Platform (http://www.omicshare.com/tools). For HCA, the normalized signal intensities of metabolites (unit variance scaling) are visualized as a color spectrum. Significantly regulated metabolites between the groups were determined by absolute Log_2_FC (fold change).

### 2.3 Transcriptome sequencing and data analysis

Total RNA was extracted using TRIzol reagent (Invitrogen, Carlsbad, CA, USA). The purity and quantification of total RNA were performed following the protocols described by (Zhang et al., 2023). An Illumina sequencing platform was used for sequencing with a read length of paired-end 150 bp. The clean reads were mapped to the reference genome (SolTub_3.0, Potato Genome Sequencing Consortium, 2011) using HISAT2. Differentially expressed genes (DEGs) were identified by comparing their raw count values using the DESeq2 package with a *p*-value < 0.05 and |log_2_FC| ≥1. The Kyoto Encyclopedia of Genes and Genomes (KEGG) analysis was performed to enrich DEG functions with webtools from the Omicshare online platform (https://www.omicshare.com/tools/).

### 2.4 Integrated metabolome and transcriptome analyses

Pearson’s correlation coefficients were calculated between the metabolome and transcriptome data. The coefficients were calculated from the log_2_FC of each metabolite and the log_2_FC of each transcript using the EXCEL program. Correlations with a coefficient of R2 > 0.95 were selected.

### 2.5 Identification of stolon- and tuber-specific gene expression

We used the transcriptomic data set of *S. tuberosum* group Tuberosum RH89-039-16, which included data for the root, stem, leaf, petiole, flower, stamen, stolon, and tuber tissues, to identify stolon- and tuber-specific genes. This analysis was performed as described previously (Yang et al., 2024).

### 2.6 Yeast one-hybrid assays

Yeast one-hybrid (Y1H) assays were performed as described previously (Yang et al., 2024). *EcoR* I and *Sal* I sites were used to insert the promoter region of 34 structural genes in the anthocyanin biosynthesis pathway into the pLacZi2u vector. Full-length *StWRKY44* was ligated into the *Xho* I and *EcoR* I sites of the GAD vector. The primers used in this study are listed in **Supplementary Table S1**. The two plasmids were co-transformed into the yeast strain EGY48. The cells were first plated onto SD-Ura/-Trp (Clontech, Shiga, Japan) selective media, and positive clones were cultured on a second selective media (SD-Ura/-Trp) supplemented with galactose, raffinose, and X-Gal.

### 2.7 Luciferase reporter assays in *N. benthamiana*

Luciferase (LUC) assays were performed as previously described (Yang et al., 2024). For the tobacco transient expression assay, the promoter region of 11 structural genes were cloned into the pGreenII0800-LUC vector using *Bsa* I, and full-length *StWRKY44* was introduced into the pGreenII62sk vector using *BamH* I and *Sal* I. The primers used were listed in **Supplementary Table S1**. Five-week-old *N. benthamiana* seedlings were used for transformation as previously described (Yang et al., 2024). Renilla and firefly luciferase activities were tested 3 days after injection by means of a Dual-Luciferase Reporter Assay Kit (Promega, Madison, USA) based on the operating instructions.

### 2.8 Real-time qPCR

Total RNA was extracted from the frozen samples using the MiniBEST Plant RNA Extraction Kit (TaKaRa, Dalian, China). We used 1 μg of total RNA for reverse transcription with ReverTra Ace qPCR RT Master Mix with gDNA Remover (TOYOBO, Osaka, Japan) following the manufacturer’s instructions. The cDNA was diluted five times before performing RT-qPCR. GoTaq qPCR Master Mix (Promega, Madison, USA) and an Applied Biosystems 7500 real-time PCR system were used to perform RT-qPCR. The primers used were listed in **Supplementary Table S1**. *StACTIN8* was used as a reference gene (Nicolas et al., 2022). Three technical replicates were used for each of the three biological replicates. The relative gene expression was calculated using the 2^–△△^ *^Ct^* method.

## 3 Results

### 3.1 Overview of the metabolome profile in purple tubers

We employed a targeted database comprising 108 compounds associated with anthocyanin metabolism to fully understand the chemical basis underlying anthocyanin accumulation in potato tubers. Anthocyanin metabolome data were generated through LC-tandem MS (LC-MS/MS)-based metabolic profiling of pith and vasculature tissues from tubers of three different potato cultivars: Qicai (e.g., purple skin, white flesh, and purple vasculature), Mila (e.g., yellow skin and yellow flesh), and Heijingang (e.g., purple skin and purple flesh) (**Figure 1A**). Three biological replicates, each consisting of pooled samples from at least three plants, were established. A total of 36 anthocyanin metabolism-related compounds were identified in at least one sample, including 25 anthocyanin monomers (7 petunidins, 7 cyanidins, 5 delphinidins, 4 pelargonidins, and 2 malvidins) and 11 flavonoids (**Supplementary Table S2**). These compounds were subsequently quantified using standard curve-based calibration to determine their absolute content and were used for further analysis. Principal component analysis (PCA) clustering was then performed to overview sample distribution, suggesting a clear divergence correlated with anthocyanin composition and content. The non-purple samples, Mila pith (MP), Mila vasculature (MV), and Qicai pith (QP), were grouped into one cluster. In contrast, the purple samples, Qicai vasculature (QV), formed a distinct cluster; Heijingang pith (HP) and Heijingang vasculature (HV) were grouped into another separate cluster (**Figure 1B**).

### 3.2 Metabolites that contributed to the purple pigmentation of the tuber

Analysis of the 25 anthocyanin monomers across different potato varieties and tissues revealed the identification of 22 anthocyanin monomers in QV, 5 in QP, 18 in HV, 17 in HP, and 4 each in MV and MP (**Figure 1C**). Among the 25 anthocyanin monomers examined, 6 anthocyanin monomers identified in MV, MP, and QP may not be associated with the purple coloration of the tubers. In contrast, the 19 anthocyanin monomers detected in HV, HP, and QV were consistent with the purple phenotype of potato tubers. Notably, five anthocyanin monomers were specific to QV, one was unique to HP, and nine were found in HV, HP, and QV (**Figure 1D**).

Furthermore, we analyzed the contents of 25 anthocyanin monomers. We found that cyanidin-type anthocyanins were significantly higher in QV, whereas the anthocyanins in HV and HP were predominantly composed of delphinidin and petunidin types (**Figure 1E**). These results indicate that although Qicai and Heijingang tubers exhibit purple coloration, the types of anthocyanins present in the two cultivars are distinct. Moreover, the contents of cyanidin-3-O-galactoside and petunidin-3-O-5-O-(6-O-coumaroyl)-diglucoside were significantly higher in HP than in HV, whereas pelargonidin-3-O-sambubioside was significantly more abundant in HV compared to HP. This suggests that, despite being derived from the same cultivar, anthocyanin content can vary among different tissues. Additionally, we identified high levels of procyanidine A1, B1, and B3 in HV, and elevated levels of rutin and naringenin in QV (**Figure 1E**). Rutin and naringenin are important flavonoids that serve as upstream precursors in anthocyanin biosynthesis. These results indicate that, although Qicai and Heijingang potato tubers exhibit purple coloration, there is a significant difference in the types and contents of anthocyanins present.

### 3.3 Comparative transcriptome analysis

We constructed a transcriptome for QV, QP, HV, HP, MV, and MP to investigate the transcriptional regulatory mechanisms underlying anthocyanin metabolism in potatoes. Approximately 263 Gb of clean data were generated, with Q20 and Q30 values of approximately 97% and 93%, respectively (**Supplementary Table S3**). On average, about 82.93% of the reads were uniquely mapped. The comparison of expression values among the three biological replicates revealed a high degree of correlation. Consequently, the average FPKM values across the three replicates were calculated to represent the gene expression levels in each sample. To minimize the impact of transcriptional noise, genes with FPKM values < 0.5 were classified as not expressed. A total of 17,043 genes were identified as expressed in at least one sample (**Supplementary Table S4**). To validate the reliability of the transcriptomic data, RT-qPCR analysis was performed on two previously published structural genes (*F3’5’H* and *F3H*) and one transcription factor (*MYB76*) associated with anthocyanin biosynthesis, along with three randomly selected genes. The results demonstrated that the expression trends of these six genes were consistent with the transcriptomic data (**Supplementary Figure S1**). Moreover, the expression patterns of *F3’5’H*, *F3H*, and *MYB76* aligned with the purple tuber phenotype. These findings indicate that the transcriptome sequencing data exhibit high accuracy in genome alignment, good reproducibility, and high reliability, accurately reflecting gene expression profiles across different tubers and tissue types.

**Figure 2:**
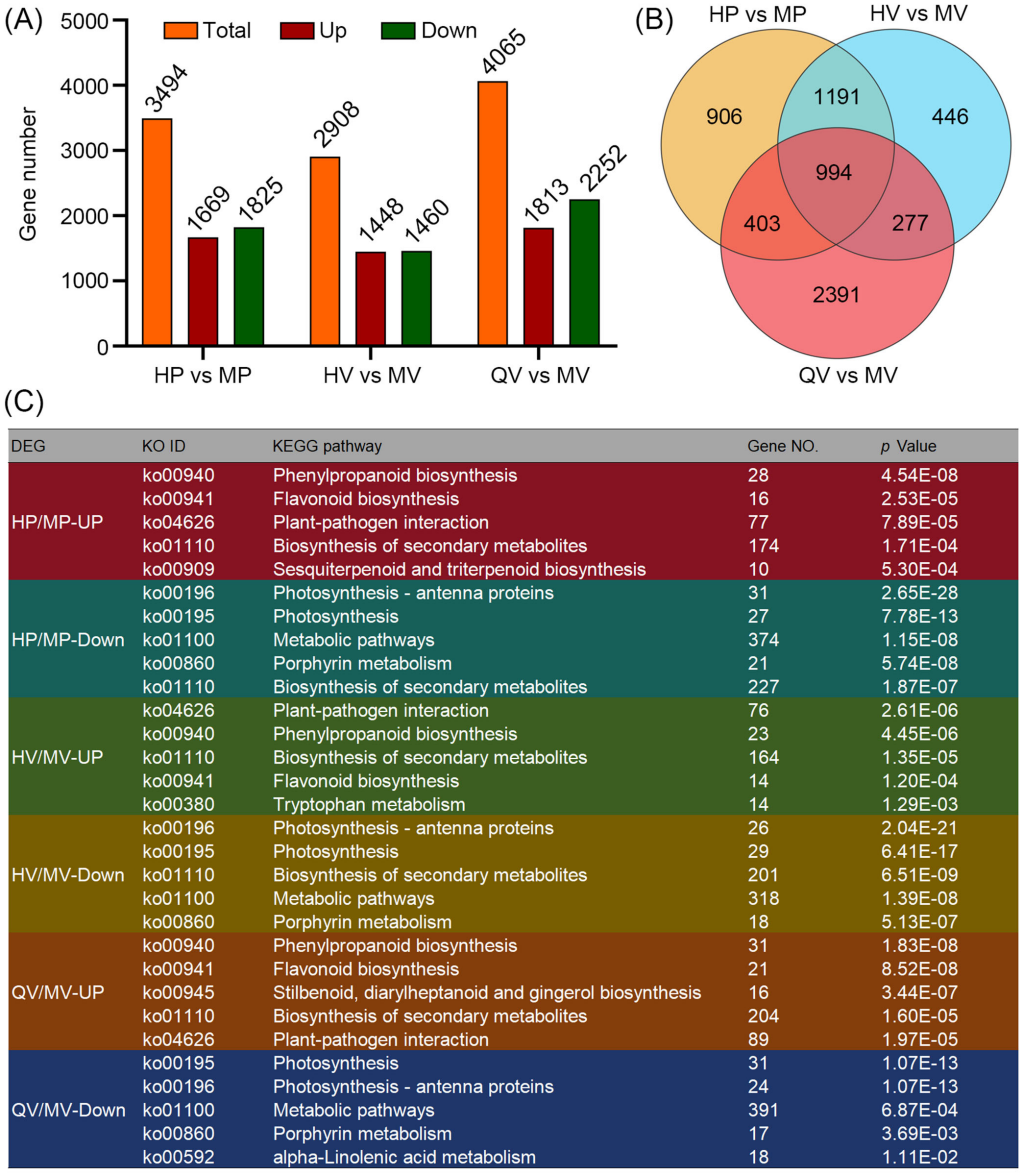
Comparative transcriptome analysis. **(A)** The column diagram shows the number of differentially expressed genes (DEGs) with up- and downregulated expression. **(B)** Venn diagram showing DEGs among HP vs. MP, HV vs. MV, and QV vs. MV. **(C)** Kyoto Encyclopedia of Genes and Genomes (KEGG) enrichment of DEGs related to anthocyanin deposition.

We identified a total of 6,608 DEGs across the three comparison groups (**Supplementary Table S5**). Specifically, there were 3,494 DEGs in the comparison of HP vs. MP, 2,908 in HV vs. MV, and 4,065 in QV vs. MV. Among these comparison groups, 1,669, 1,448, and 1,813 genes were upregulated, while 1,825, 1,460, and 2,252 genes were downregulated, respectively (**Figure 2A**). Notably, 994 DEGs were common in all three comparison groups (**Figure 2B**). We subjected 6,608 DEGs to KEGG pathway enrichment and found that the upregulated DEGs across the three comparison groups were predominantly enriched in “phenylpropanoid biosynthesis” (ko00940), “flavonoid biosynthesis” (ko00941), and “plant–pathogen interaction” (ko04626). In contrast, the downregulated DEGs were primarily enriched in “photosynthesis” and “porphyrin metabolism” (**Figure 2C**). These findings suggest that despite variations in anthocyanin content and types among different materials and tissues, the functional roles of the genes implicated in anthocyanin biosynthesis remain relatively consistent. Thus, these DEGs are likely to play major roles in the synthesis of anthocyanins.

### 3.4 Expression patterns of genes involved in anthocyanin biosynthesis and metabolism

**Figure 3:**
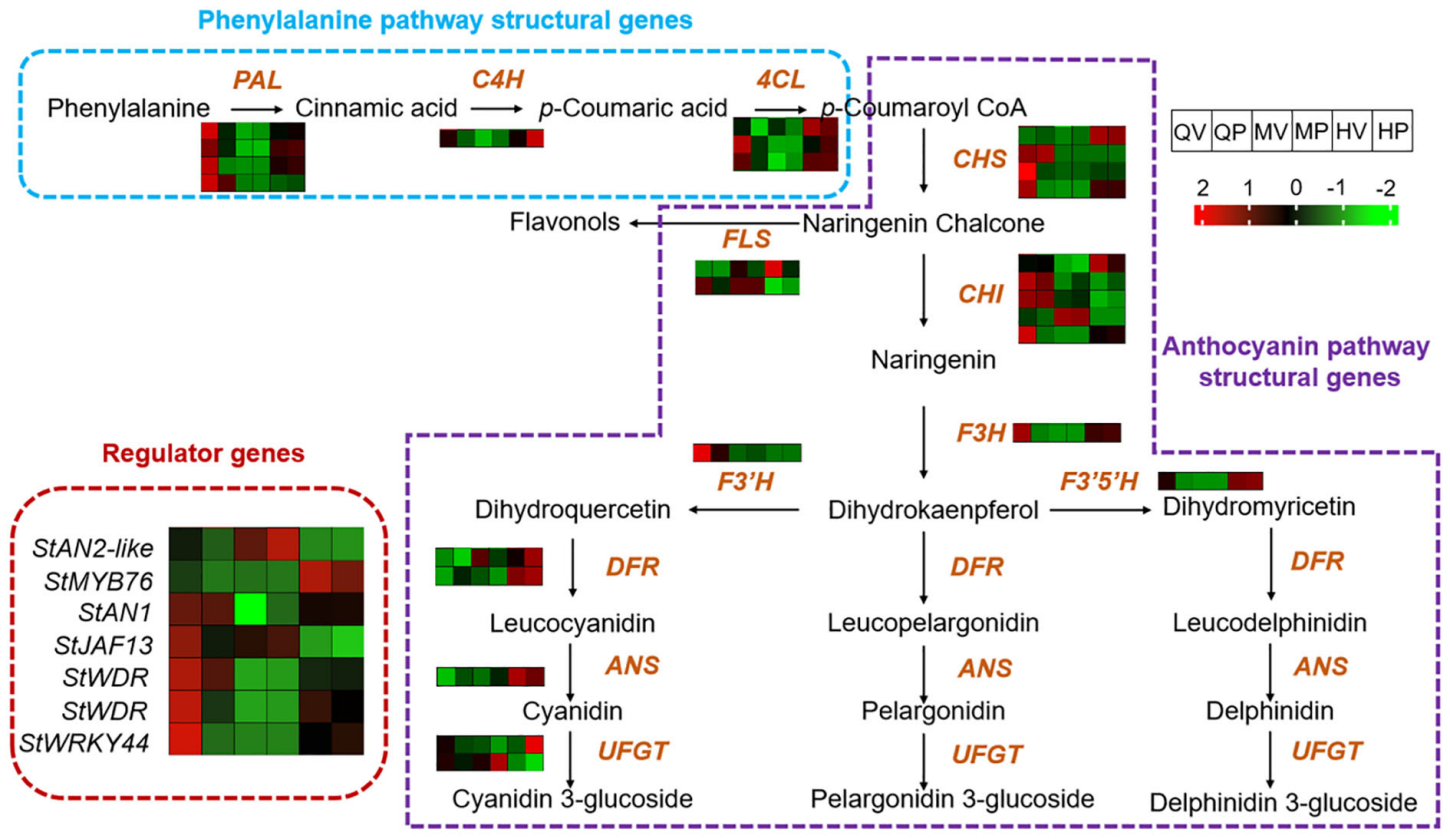
Expression of structural genes and regulatory genes in the anthocyanin biosynthesis pathway. For each gene, the normalized expression values (FPKM) were calculated by the maximum value of all gene expression levels for six different samples. The color scale was normalized to range from −2 (green) to +2 (red), which represented the high and low expression values, respectively.

We analyzed the transcript levels of 34 candidate structural genes and 7 regulatory genes in relation to anthocyanin biosynthesis (**Supplementary Table S6**) (Zhu et al., 2017; Sun et al., 2020a; Zhang et al., 2024). Located upstream in the pathway, from phenylalanine to *p*-Coumaroyl CoA, four *PALs*, one *C4H*, and three *4CLs* were significantly upregulated in QV, HV, and HP compared with QP, MV, and MP (**Figure 3**). This result suggests that the expression patterns of upstream genes involved in anthocyanin biosynthesis are largely consistent between Qicai and Heijingang.

We then compared the expression of genes involved in the conversion from *p*-Coumaroyl CoA to naringenin. Among the identified homologs, four *CHSs* and five *CHIs* were identified. However, only one *CHS* gene and one *CHI* gene were upregulated in QV, HV, and HQ. One *CHS* gene was specifically upregulated in QV, while another *CHS* gene exhibited specific upregulation in HV and HQ. The expression patterns of the remaining genes showed no correlation with anthocyanin content in the tuber (**Figure 3**). These findings indicate that the genes involved in the biosynthesis of anthocyanins from *p*-Coumaroyl CoA to naringenin have undergone significant changes in QV, HV, and HP.

**Figure 4:**
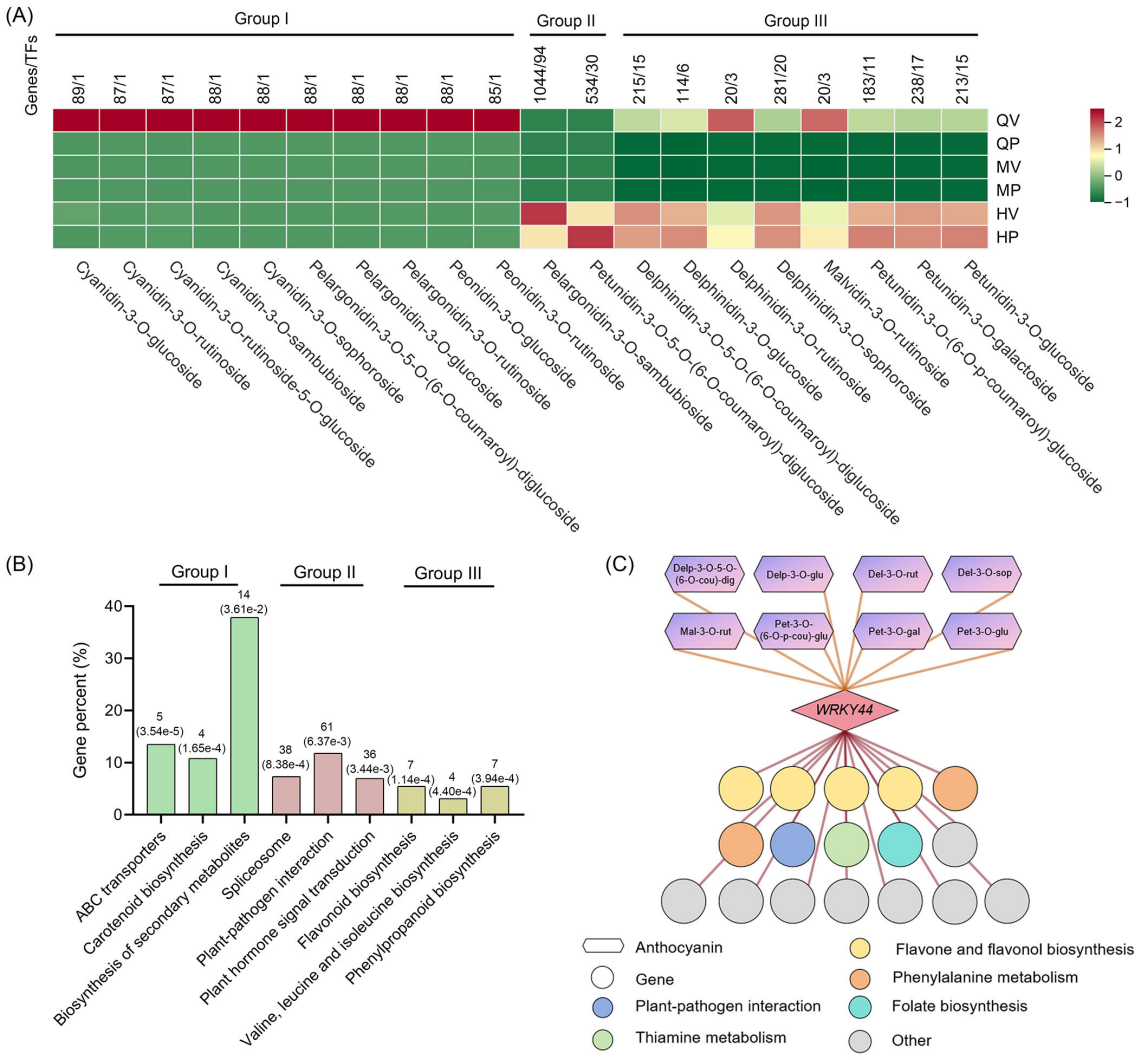
Expression patterns of genes involved in anthocyanin biosynthesis. **(A)** Association analysis between genes and 20 anthocyanins related to the purple phenotype of tubers. Pearson’s correlation coefficient was greater than 0.95. P-values lower than 0.01. One thousand nine hundred twenty-four genes were clustered into three groups corresponding to different anthocyanins. **(B)** Kyoto Encyclopedia of Genes and Genomes (KEGG) analysis of groups identified as correlated with different anthocyanins. The ordinate shows the ratio of the genes annotated to the total number of genes annotated. The horizontal axis represents the name of the KEGG pathway. The numbers within brackets represent *P*-values. **(C)** The coexpression network of *StWRKY44* is involved in the anthocyanin biosynthesis of tubers in potatoes. Circles represent genes, with different colors indicating annotations in various KEGG pathways; hexagons denote different anthocyanins; and rectangles represent the transcription factor *StWRKY44*.

*F3H* catalyzes the conversion of naringenin into dihydroflavonols and is responsible for the biosynthesis of flavonols and anthocyanidins (Winkel-Shirley, 2001). In potatoes, there is a single homologous gene for *F3H*, which exhibits extremely high expression levels in QV, HV, and HP. *F3′H* and *F3′5′H* converted dihydrokaempferol into different pathways, producing cyanidin or delphinidin and petunidin, respectively. We identified one homolog each of *F3′H* and *F3′5′H*. Notably, *F3′H* was significantly upregulated in QV, while *F3′5′H* exhibited significant upregulation in HV and HQ (**Figure 4**). These findings are consistent with the metabolomics results mentioned above, indicating that the high content of cyanidins in QV may be linked to the *F3′H* gene, and *F3′5′H* may play a pivotal role in enhancing the metabolic flux toward the accumulation of delphinidins and petunidins (Winkel-Shirley, 2001). In addition, two *DFR* genes and one *ANS* gene were highly expressed in QV and QP. At the same time, one *UFGT* gene was highly expressed in QV and HP. Moreover, the expression of several anthocyanin regulatory genes was examined. *MYB76* performed significant upregulation in HV and HP, and *StWRKY44* was consistently upregulated in QV, HV, and HP. This result suggests that anthocyanin-related TFs with different expression patterns may play a role in regulating the accumulation of different anthocyanins in various tissues of the tuber.

### 3.5 Identification of genes controlling anthocyanin biosynthesis in tubers

We conducted an association analysis of 20 anthocyanins related to the purple phenotype of tubers to identify genes that exhibited a high correlation with these anthocyanins. A total of 1,924 (Pearson’s coefficient greater than 0.95, P-values lower than 0.01) genes were identified, including 145 TFs (**Supplementary Table S7**). These 1,924 genes were clustered into three groups corresponding to different anthocyanins. Group I (98 genes, 1 TF) included 10 anthocyanins, with genes primarily exhibiting high expression in QV; Group II (1,576 genes, 124 TFs) contained 2 anthocyanins, with gene expression patterns correlating with anthocyanin accumulation in HV and HP; and Group III (346 genes, 25 TFs) comprised 8 anthocyanins, with genes showing elevated expression in QV, HV, and HP (**Figure 4A**). We performed KEGG pathway enrichment to assign genes to functional categories for each group to further provide insights into the function of the genes associated with the different anthocyanins (**Figure 4B**).

Genes expressed in QV (Group I). In Group I, genes related to ATP-binding cassette (ABC) transporters, including homologous genes already reported in Arabidopsis, such as *ABC15B*, *ABCG11*, *ABCG8*, *ABCG22*, and *ABCG5*. These ABC transporters mediate the movement of monomers and phytohormones across diffusion barriers (Do et al., 2018). Additionally, four genes encoding NRT1/PTR FAMILY transporter proteins were identified in Group I. These NRT1/PTR transporters are capable of transporting multiple substrates, such as nitrite, chloride, glucosinolates, auxin (IAA), abscisic acid (ABA), jasmonates (JAs), and gibberellins (GAs) (Corratgé-Faillie and Lacombe, 2017). These transport-related genes will be helpful for further understanding the mechanism underlying the specific accumulation of anthocyanins in the tuber vasculature. Furthermore, Group I contains only one TFs that is homologous to *AST1* from the Trihelix family in Arabidopsis. Increased expression of *AST1* has been shown to eliminate the accumulation of reactive oxygen species in cells (Xu et al., 2018). Given that anthocyanins in plants possess strong antioxidant and free radical scavenging potential, it is hypothesized that this TFs may be a key regulator of anthocyanin accumulation in the tuber vasculature.

Genes expressed in HV and HP (Group II). In Group II, genes were enriched in the plant hormone signal transduction and plant–pathogen interaction pathways. Plant hormones play a critical role in regulating anthocyanin biosynthesis, as ABA, IAA, and JA all promote anthocyanin accumulation in plants (An et al., 2021a, 2021b; Wang et al., 2018). In this group, 36 genes involved in the plant hormone signal transduction pathway were identified, including 5 IAA-related genes, such as the previously published *IAA13*, *SAUR64*, and *SAUR50*; 4 JA-related genes, including the well-documented *JAZ1* and *JAZ3*; and 4 ABA-related genes, such as *ABA2*, *AREB3*, and *PYL8*. These results reflect the importance of plant hormone genes in anthocyanin biosynthesis in HV and HP. Plant disease resistance is also closely linked to anthocyanin accumulation. Plant resistance is accompanied by higher expression of defense and anthocyanin genes at early stages of infection (Mewa et al., 2023; Zhang et al., 2019), and 46 genes involved in the plant–pathogen interaction pathway were identified in Group II. Exploring the function of these defense-response-related genes might be useful for understanding the defense-response mechanism of anthocyanins in plant resistance against pathogen infection.

Additionally, we identified 38 genes involved in the spliceosome pathway. Alternative splicing greatly expands proteome diversity and can significantly affect protein accumulation and regulation (Baralle and Giudice, 2017). In Group II, we identified the splicing factor SKIP, which binds to the pre-mRNA of the ABA signaling-related gene *PYL8* (also identified in this group) to regulate its splicing and activate its expression (Zhang et al., 2022). The analysis reveals the importance of spliceosome components in anthocyanin biosynthesis.

Genes expressed in QV, HV, and HP (Group III). The genes in Group III were significantly enriched in the flavonoid biosynthesis and phenylpropanoid biosynthesis pathways. Anthocyanins were synthesized through the flavonoid branch of the general phenylpropanoid pathway. Enhanced expression of anthocyanin biosynthesis genes leads to substantial upregulation of genes within the flavonoid and phenylpropanoid biosynthesis pathways (Sun et al., 2020a). Additionally, several structural genes involved in anthocyanin biosynthesis were identified in Group III, including *F3H*, *F3’H*, *CHI*, *ACC*, *CHS*, and *PAL*. Network analysis of coexpression regulation for the core TFs *WRK44* in Group III revealed that its predicted downstream target genes are also associated with the flavonoid and phenylpropanoid biosynthesis pathways (**Figure 4C**).

Moreover, the genes in Group III are also related to the valine, leucine, and isoleucine biosynthesis pathways. Leucine, isoleucine, and valine are classified as branched-chain amino acids (BCAAs). BCAAs are important signaling molecules in animals; however, their roles in plant growth and development remain largely unexplored (Sun et al., 2020b; Valerio et al., 2011; Xing and Last, 2017). Investigating the relationship between anthocyanins and BCAAs may provide further insights into the molecular mechanisms underlying anthocyanin biosynthesis.

### 3.6 Tuber-specific genes regulate anthocyanin accumulation in potato tubers

We utilized a previously published dataset of 782 tuber-specific expressed genes (Yang et al., 2024) to further investigate the tissue-gene relationship underlying the specific synthesis and accumulation of anthocyanins in potato tuber and identify genes that are exclusively expressed in the tubers across Groups I–III. The results revealed that there are 6, 36, and 5 tuber-specific expressed genes in Groups I, II, and III, respectively (**Supplementary Table S8**). We identified two ABC transporter G family genes, *ABCG5* and *ABCG8*, in Group I. Additionally, two genes involved in the ubiquitin carboxyl-terminal hydrolase pathway were identified in this group. Ubiquitin carboxyl-terminal hydrolases belong to a subclass of deubiquitylating enzymes that not only help generate and maintain the supply of free ubiquitin monomers but also directly modulate the functions and activities of specific target proteins (Hayama et al., 2019). These genes may play a crucial role in the specific accumulation of anthocyanins in potato tuber vasculature tissues. In Group II, we identified five TFs, such as *MYC3*, which regulates the JA and ETH pathway (Chico et al., 2020); *ERF13*, which regulates the IAA pathway (Lv et al., 2021); *HB40*, which regulates the GA pathway (Dong et al., 2022). The results indicate that these tuber-specific TFs may influence anthocyanin accumulation in tubers by regulating crosstalk among different plant hormones. Functional analysis of the five genes in Group III revealed the presence of the previously published TFs *BBX22*, which directly bound to and activated the expression of *HY5*; GA metabolism gene *GA2ox8*; carotenoid biosynthesis gene *PSY1*; anthocyanin regulatory gene *Ruby1*, which played a dual role in regulation of internode elongation and pigmentation (Fu et al., 2024). In summary, the tuber-specific genes identified here will be useful for understanding the specific biological processes occurring during anthocyanin synthesis and accumulation in potato tubers.

### 3.7 Validation of correlation analyses

**Figure 5:**
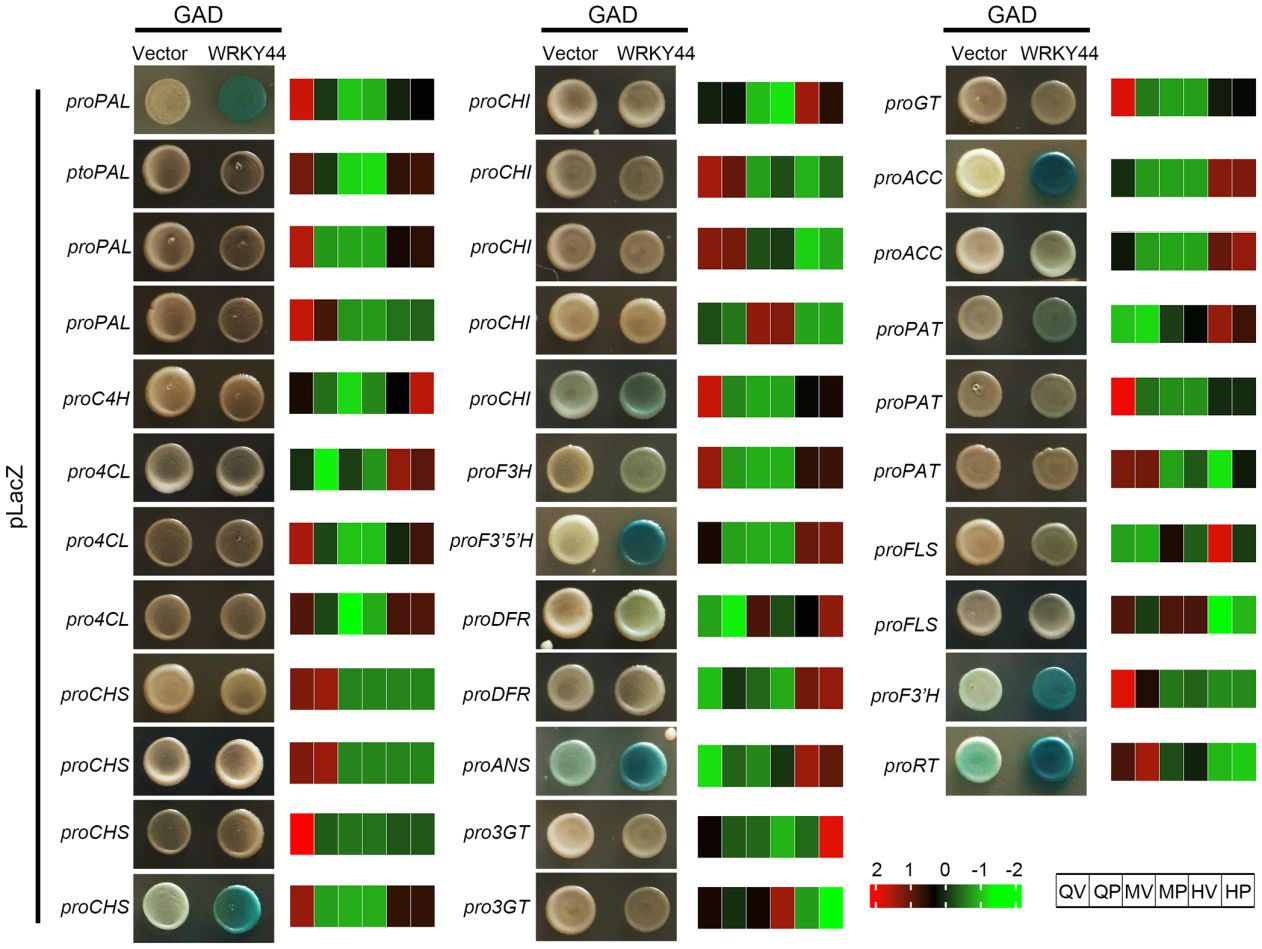
Yeast one-hybrid (Y1H) assays of transcription factor (TF) *StWRKY44* and 34 structural genes regulations. Valid binding between *StWRKY44* and a gene promoter was verified only if the test exhibited a blue color and the respective negative control showed no such blue color. For each gene, the normalized expression values (FPKM) were calculated by the maximum value of all gene expression levels for six different samples. The color scale was normalized to range from −2 (green) to +2 (red), which represented the high and low expression values, respectively.

To further validate the accuracy and reliability of the gene correlation analysis, we selected the core TFs *StWRKY44*, identified in Group III. Phylogenetic analysis revealed that the candidate StWRKY44 gene is closely related to Arabidopsis AtWRKY44/TTG2 (**Supplementary Figure S2A**), which is a WRKY TF that regulates proanthocyanidin synthesis in the seed coat (Gonzalez et al., 2016). A multiple protein sequence alignment indicated that StWRKY44 contains C2H2-type zinc finger motifs and two WRKY domains, which are conserved in the WRKY44 proteins of other species (**Supplementary Figure S2B**). Then we employed Y1H assays to assess the binding affinity of StWRKY44 to the promoters of 34 structural genes in the anthocyanin biosynthesis pathway. The results revealed that StWRKY44 binds to the promoters of 11 genes, including *PAL*, *CHI*, *CHS*, *F3H*, *DFR*, *ANS*, *F3’5’H*, *ACC*, *PAT*, *F3’H*, and *RT* (**Figure 5**). To further confirm if 11 structural genes expression were activated by StWRKY44, an transient promoter activation assays were performed in *N. benthamiana*. The dual luciferase assay showed that StWRKY44 was able to strongly activate the *CHS*, *CHI*, *F3H*, *F3’5’H*, *DFR*, *ANS*, *F3’H* promoters, with a significant increase in the LUC-to-Renilla (LUC/REN) ratio compared with the promoter-only control (**Supplementary Figure S3**). Among them, the expression patterns of *CHS*, *CHI*, *F3H*, and *F3’5’H* are similar to those of *StWRKY44*, indicating that the transcriptomic data and correlation analysis are accurate and reliable. Further analysis found that the structural genes *CHSs* and *CHIs* have multiple homologs in potatoes, and StWRKY44 can regulate only one of these homologs. Whether the remaining homologous genes are involved in anthocyanin accumulation in potato tubers requires further experimental analysis and validation in future studies.

## 4 Discussion

Anthocyanins contribute to the purple pigmentation of potatoes, enhancing their appeal to consumers. In addition to providing resistance against biotic and abiotic stresses, these compounds offer potential health benefits for humans, including the prevention of various chronic diseases and protection against cardiovascular diseases and certain cancers (Pojer et al., 2013). Consequently, the production of anthocyanins in colored potatoes has garnered considerable interest, particularly in elucidating the underlying regulatory mechanisms. Previous studies have explored colored potato tubers through integrated transcriptomic and metabolomic analyses (Liu et al., 2023; Zhang et al., 2023). However, differences in anthocyanin composition across various tissue regions of potato tubers, as well as the corresponding genetic regulatory networks, remain largely unexplored. In our study, we comprehensively analyzed the metabolomic and transcriptomic differences in pith and vasculature tissues from three different potato cultivars to identify candidate genes involved in anthocyanin biosynthesis. We identified 25 anthocyanin monomers, 11 flavonoid monomers, and 17,043 genes. These data provide valuable insights into the molecular basis of anthocyanin biosynthesis in potato tubers and will facilitate further elucidation of the underlying regulatory networks.

We identified 1,924 genes (including 145 TFs) that exhibited a high correlation with 20 anthocyanins by integrating transcriptomic and metabolomic datasets to analyze the genes regulating anthocyanin biosynthesis in tubers. These 1,924 genes were clustered into three groups corresponding to different anthocyanins. Genes in Group I are expressed in the QV, and these genes are functionally associated with transporter proteins. Anthocyanins are synthesized in the cytosol and transported into the vacuole for storage or to other destinations where they function as bioactive molecules. Two models explain the mechanism of anthocyanin transport. The first model was ligandin transportation. GST transporters, as a ligandin, bind cytoplasmic anthocyanins, escorting them from the endoplasmic reticulum to the tonoplast. Once at the tonoplast, anthocyanins penetrate the vacuolar membrane primarily through multidrug resistance-associated proteins, members of the ABC transporter family (Goodman et al., 2004; Jasinski et al., 2003; Mueller et al., 2000; Sun et al., 2012). The second model, vesicular transport, involves pre-vacuolar compartment vesicles that carry anthocyanins to the vacuole, with multidrug and toxic compound extrusion (MATE), or anthoMATE (AM), acting as the key transporter in this process (Gomez et al., 2011). These transport-associated genes are likely critical for elucidating the molecular mechanisms governing the spatiotemporal accumulation of anthocyanins within the tuber vasculature. In Group II, genes expressed in HV and HP showed enrichment in the plant hormone signal transduction and plant–pathogen interaction pathways. Phytohormones, including ABA, JA, ETH, SL, GA, and IAA, play pivotal roles in regulating anthocyanin biosynthesis. Phytohormones orchestrate anthocyanin biosynthesis through crosstalk with TFs. In Group II, 124 TFs were identified, and their co-regulation with hormones may play a crucial role in the accumulation of anthocyanins in HV and HP. Anthocyanins also protect plants against pathogen infection (Li et al., 2019; Mewa et al., 2023). However, the precise regulation of the mechanisms of anthocyanin involved in the plant–pathogen interaction pathway remains to be elucidated. In Group III, 346 genes were identified as being expressed in QV, HV, and HP. These genes are related to flavonoid and phenylpropanoid biosynthesis. Several structural genes involved in anthocyanin biosynthesis were also identified in this group, such as *F3H*, *F3’H*, *CHI*, *ACC*, *CHS*, and *PAL*. The data from Groups I–III provide an information-rich resource for investigating the functions of anthocyanin biosynthesis in potato tubers.

Anthocyanin biosynthesis has been extensively studied in plants, leading to the identification of numerous structural genes and TFs that regulate this pathway. In this study, we analyzed the transcript levels of 27 candidate structural genes and 7 regulatory genes related to anthocyanin biosynthesis. The expression patterns of upstream genes were largely consistent among QV, HV, and HP, whereas downstream genes, such as *DFR* and *UFGT*, exhibit differential expression across these tissues. *DFR* plays a central role in anthocyanin biosynthesis by reducing several flavonoid precursors of anthocyanins. Variations at different sites in the *DFR* coding region determine the capacity of DFR enzymes to produce different types of anthocyanin pigments (e.g., purple, red, or blue) by converting different dihydroflavonol substrates (Des Marais and Rausher, 2008; Wang et al., 2022). The UFGT enzyme represents the terminal gene in the anthocyanin biosynthetic pathway and also exhibits relatively higher non-synonymous substitution rates compared to the upstream genes (Huang et al., 2016; Rausher et al., 1999). *DFR* and *UFGT* have two homologous genes in potatoes, and differences in their sequences and expression patterns potentially lead to the synthesis of distinct anthocyanins in the tubers.

Furthermore, we observed that *F3’H* was highly expressed in QV. *F3’H* is a microsomal cytochrome P450-dependent monooxygenase that catalyzes the NADPH-dependent hydroxylation of substrates (Awasthi et al., 2016). It is critical to determine the hydroxylation pattern of the anthocyanin aglycone, resulting in the accumulation of cyanidin-based anthocyanins (Peng et al., 2020). In kiwifruit, peanut, Chinese cabbage, and other plants, the expression of F3′H was strongly correlated with the content of different flavonoids, respectively (Park et al., 2021; Peng et al., 2019; Xue et al., 2021). Therefore, it is speculated that the expression pattern of *F3’H* may be related to the accumulation of anthocyanins in QV.

Some potato cultivars produce anthocyanins in response to exogenous biotic or abiotic stresses; however, these anthocyanins are primarily synthesized and accumulated in the leaves and stems, with minimal deposition in the tubers (Strygina et al., 2019; Kaur et al., 2023). Therefore, identifying tuber-specific anthocyanin-related genes is of great significance for elucidating why anthocyanins accumulate in potato tubers. In this study, we identified six tuber-specific expression of anthocyanin-related transcription factors, which primarily function in hormone-related pathways. For instance, MYC3 regulates ETH and JA pathways (Chico et al., 2020), ERF13 modulates the IAA pathway (Lv et al., 2021), and HB40 influences the GA pathway (Dong et al., 2022). Additionally, ERF13 and HB40 are involved in the regulation of lateral branching (Lv et al., 2021; Dong et al., 2022), which is closely associated with tuber formation in potato. Based on these findings, we hypothesize that these transcription factors may promote tuber development by modulating hormonal pathways and concurrently regulate anthocyanin accumulation in the tubers.

*StWRKY44* is highly similar to *Arabidopsis TTG2*, which participates in trichome development and controls seed coat tannins by regulating the expression of the vacuolar transporter of glycosylated epicatechin (Gonzalez et al., 2016). In pear, *PpWRKY44* promoted anthocyanin accumulation by activating *PpMYB10* and was regulated by *PpBBX18*, a light signal transduction pathway component (Alabd et al., 2022). In Asiatic hybrid lilies, *LhWRKY44* activated and bound to the promoters of gene *LhF3H* and t *LhGST* (Bi et al., 2023). In kiwifruit, *WRKY44* was able to strongly activate the promoters of the kiwifruit *F3′H* and *F3′5′H* genes (Peng et al., 2020). In our study, we found that *StWRKY44* was the hub gene in Group III and could bind to the promoters of 11 structural genes, including *PAL*, *CHI*, *CHS*, *F3H*, *DFR*, *ANS*, *F3’5’H*, *ACC*, *PAT*, *F3’H*, and *RT*. This result indicates that *StWRKY44* may play a major role in the regulation of multiple structural genes involved in various anthocyanin biosynthetic processes, further suggesting that *StWRKY44* could be an important regulatory gene in anthocyanin synthesis in potatoes.

## 5 Conclusion

In conclusion, we performed metabolomic and transcriptomic analyses to explore the chemical and genetic foundations of anthocyanin in potato tubers, and identified 20 anthocyanins as key metabolites responsible for the purple pigmentation in tubers, which were correlated with 1,924 genes associated with ABC transporters and phytohormones. Additionally, we identified a core transcription factor, *StWRKY44*, that is involved in anthocyanin accumulation within the tubers; this transcription factor was found to bind to and activate the promoters of 7 anthocyanin structural genes. Functional studies should be performed in the future to validate their role in the regulation of anthocyanin biosynthesis in potato tubers. Our study provides insights into the genes and metabolites associated with anthocyanin accumulation in tubers, paving the way for future functional studies and advancements in potato breeding.

## Data Availability

The raw sequence data reported in this paper are publicly available in the Genome Sequence Archive (Genomics, Proteomics & Bioinformatics 2021) in National Genomics Data Center (Nucleic Acids Res 2024) under project accession PRJCA041167 (https://ngdc.cncb.ac.cn/gsa).
