# Supplementary material for: Transcriptomic and targeted metabolomic analysis identifies genes involved in differential anthocyanin accumulation in potato tubers
Source: Front Plant Sci. 2025 Jun 19;16:1615972. doi: 10.3389/fpls.2025.1615972 (PMC12221910; doi:10.3389/fpls.2025.1615972)
Supplement: Supplementary file 1 [file Image1.pdf]

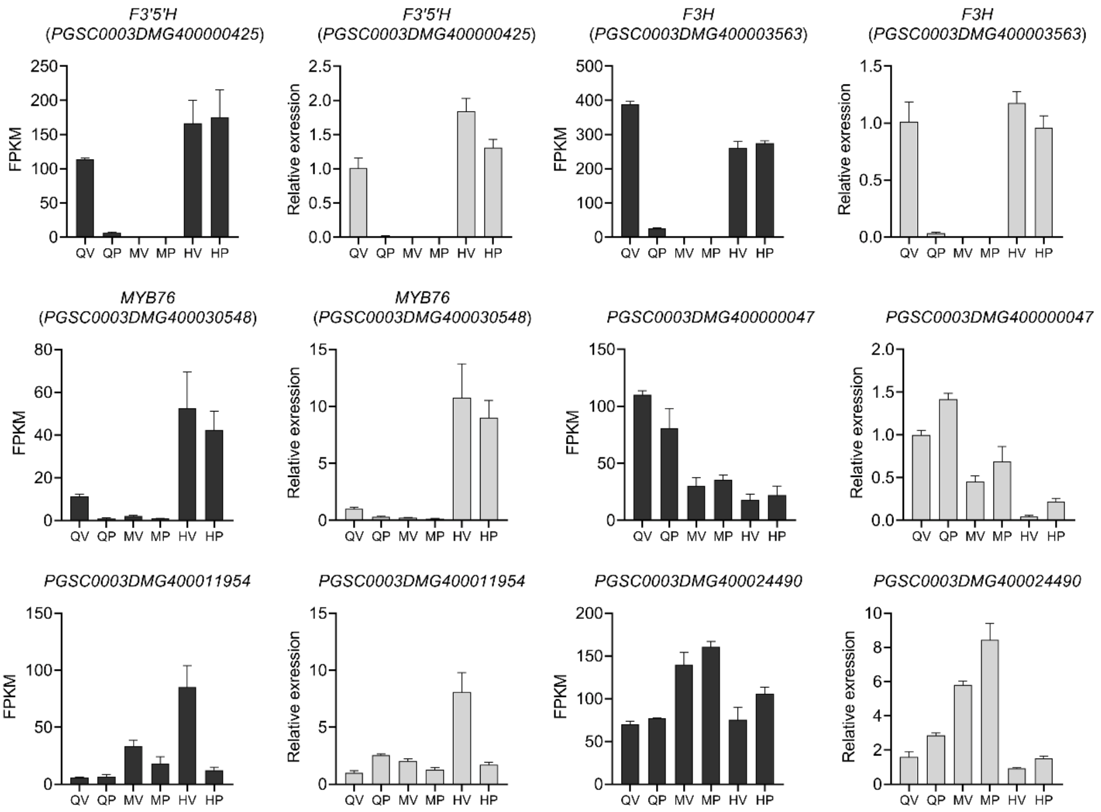

Figure S1. Validation of transcriptomic genes by RT-qPCR. Three independent biological repetitions were performed in each experiment. *StACTIN8* was used as a reference gene. All the data represent the mean ± SD.

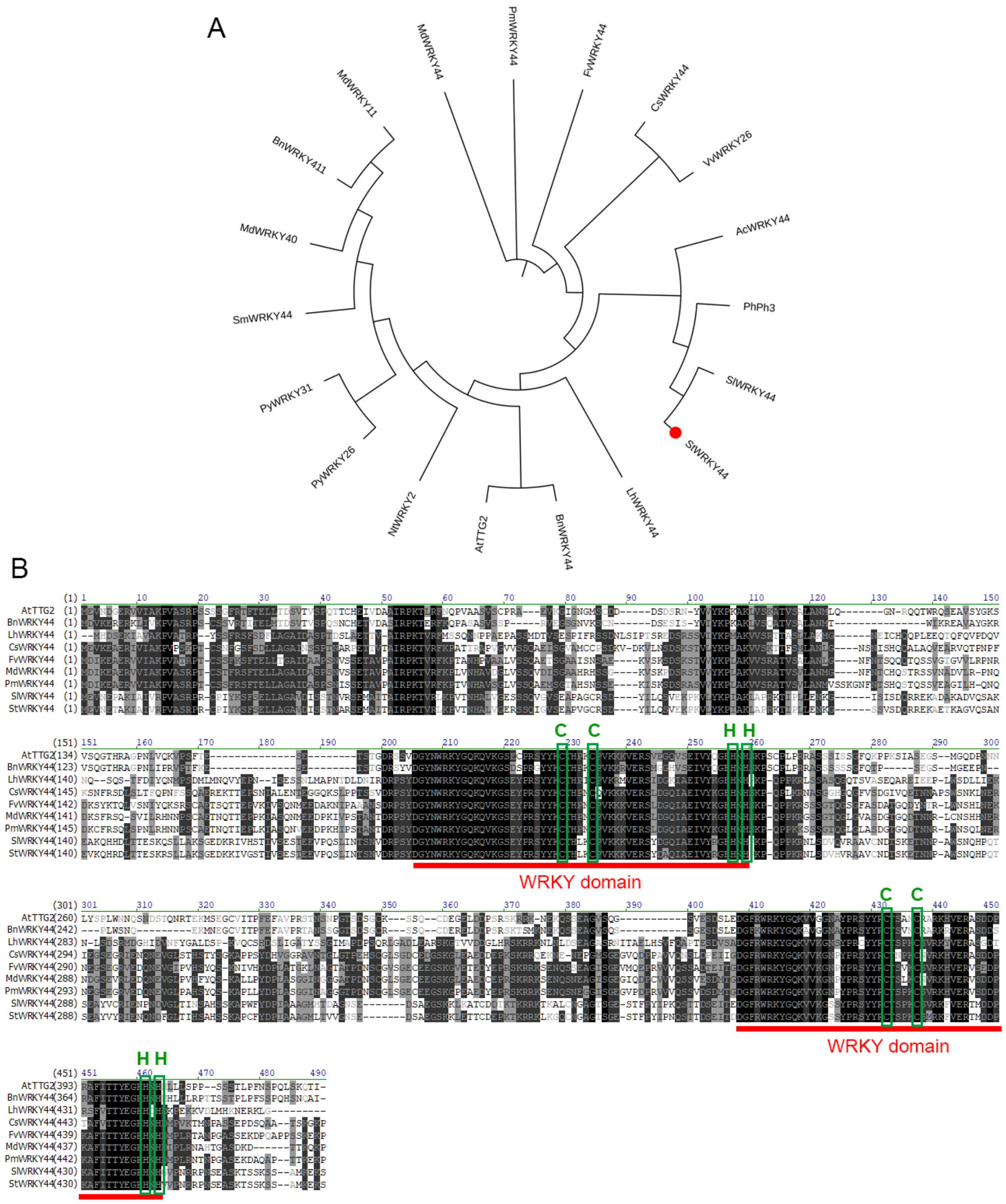

Figure S2. Sequence characteristics of StWRKY44. (A) Phylogenetic relationship of StWRKY44 and other WRKYs from other species conducted on the basis of protein sequences. StWRKY44 is marked by a red circle. *Ac*, *Actinidia sp.* (AcWRKY44, ACC16887.1); *At*, *Arabidopsis thaliana* (AtTTG2, NP_181263.2); *Bn*, *Brassica napus* (BnWRKY41-1, XP_013686534; BnWRKY44-2, XP_022557932.1); *Cs*, *Camellia sinensis* (CsWRKY44, AYA73391); *Fv*, *Fragaria vesca* (FvWRKY44, XP_004302832.1); *Lh*, *Lilium spp.* (LhWRKY44, UYP11329.1); *Md*, *Malus domestica* (MdWRKY11, MDP0000128463; MdWRKY40, XP_008342807; MdWRKY44, XP_008387690.2); *Nt*, *Nicotiana tabacum* (NtWRKY2, BAB61056.1); *Ph*, *Petunia hybrida* (PhPh3, AMR43368); *Pm*, *Prunus mume* (PmWRKY44, XP_008242029.1); *Py*, *Pyrus* L. (PyWRKY26, Pbr013092; PyWRKY31, Pbr000122); *Sl*, *Solanum lycopersicum* (SlWRKY44, XP_004249802.1); *Sm*, *Salvia miltiorrhiza* (SmWRKY44, AKA27910.1); *St*, *Solanum tuberosum* (StWRKY44,

XP_XP_006339294.2); *Vv*, *Vitis vinifera* (VvWRKY26, AQM37647). (B) Sequence alignment of StWRKY44 and other WRKY transcription factors. Red lines represent the conserved WRKY amino acid domains, whereas green letters represent zinc finger motifs.

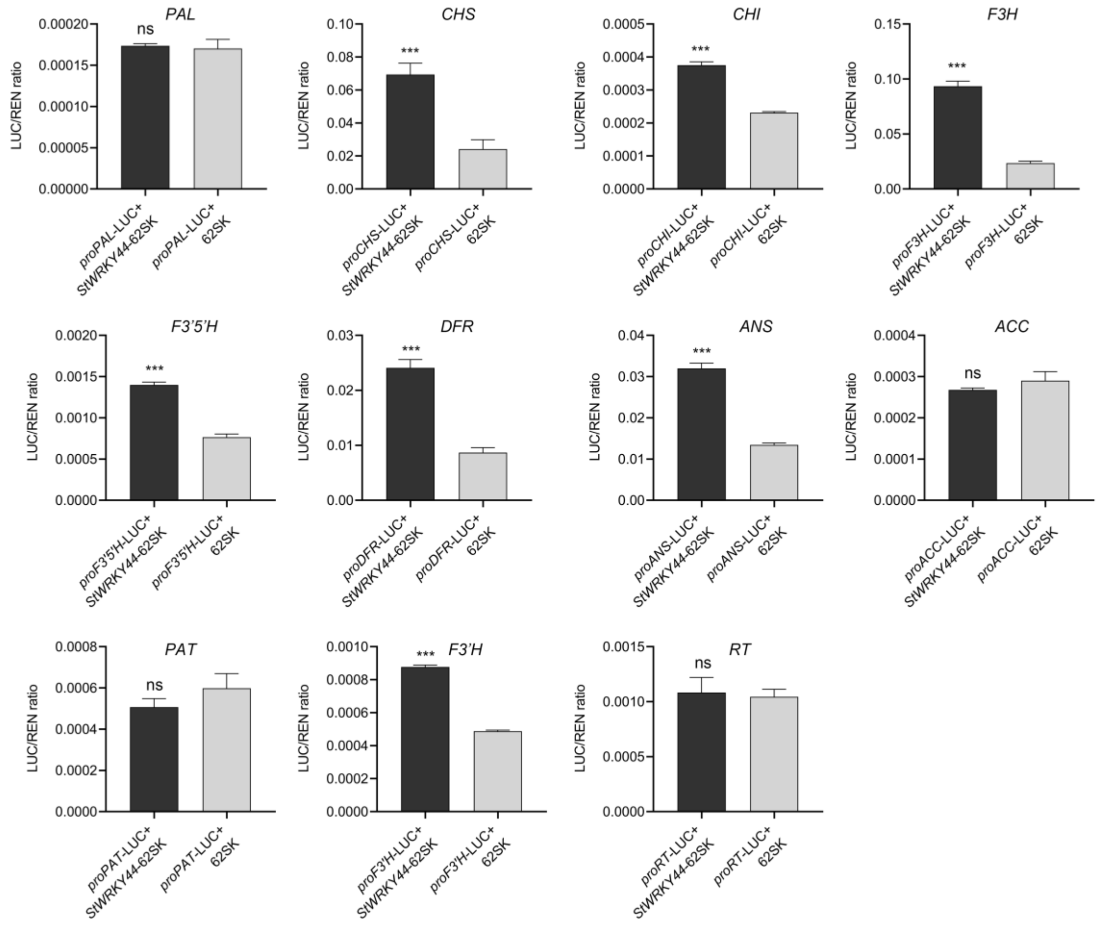

Figure S3. Luciferase (LUC) activation assay. *StWRKY44* transient expression in *N. benthamiana* leaves. The promoter sequence of 11 structural genes was inserted into a LUC vector (pGreenII0800-LUC). The 62SK vector (pGreenII62sk) represents the empty vector control. The relative expression of *pro::LUC* was normalized to that of *35S::REN* (internal control) (LUC/REN, mean ±SD, n=3). All the asterisks in this figure indicate a statistically significant difference (Student's *t* test, ***P<0.01). ns means no significant difference.
